# The Medical Student's Vade Mecum

**Published:** 1852-04

**Authors:** 


*The Medical Student's Vade Mecum.* By GEORGE MENDENHALL, M. D. Third edition, with two hundred and twenty-four engravings. PHILADELPHIA: LINDSAY & BLAKISTON, 1852, pp. 690.

THIS excellent epitome is very naturally popular among students. It furnishes a compend of medical science in the form of questions and answers, and cannot but be advantageous to those who use it. Young practitioners may also find it very useful. Like all of Lindsay & Blakiston's books, it is well printed, and the cuts are good. These publishers evidently spare no expense to furnish a good article, and the works from their press should be proportionably encouraged.

